# Association of *Trypanosoma cruzi* infection with risk factors and electrocardiographic abnormalities in northeast Mexico

**DOI:** 10.1186/1471-2334-14-117

**Published:** 2014-03-01

**Authors:** Zinnia Judith Molina-Garza, José Luis Rosales-Encina, Roberto Mercado-Hernández, Daniel P Molina-Garza, Ricardo Gomez-Flores, Lucio Galaviz-Silva

**Affiliations:** 1 Universidad Autónoma de Nuevo León, Facultad de Ciencias Biológicas, Ave. Universidad SN, Cd. Universitaria, San Nicolás de los Garza, Nuevo León 66451, México; 2 Departamento de Infectómica y Patogénesis Molecular, CINVESTAV, Av. Instituto Politécnico Nacional 2508, Col. San Pedro Zacatenco, Del. Gustavo A. Madero, México City, CP 07360DF, México; 3 Secretaría de Salud del Estado de Nuevo León, Hospital General de Cerralvo, Cerralvo, Nuevo León 66451, México

**Keywords:** Chagas disease, *Trypanosoma cruzi*, Serology, Electrocardiographic abnormalities, Epidemiology

## Abstract

**Background:**

American trypanosomiasis is a major disease and public health issue, caused by the protozoan parasite *Trypanosoma cruzi*. The prevalence of *T. cruzi* has not been fully documented, and there are few reports of this issue in Nuevo Leon. The aim of this study was to update the seroprevalence rate of *T. cruzi* infection, including an epidemiological analysis of the risk factors associated with this infection and an electrocardiographic (ECG) evaluation of those infected.

**Methods:**

Sera from 2,688 individuals from 10 municipalities in the state of Nuevo Leon, Mexico, were evaluated using an enzyme-linked immunosorbent assay and an indirect hemagglutination assay. An ECG case–control study was performed in subjects seropositive for *T. cruzi* and the results were matched by sex and age to seronegative residents of the same localities. A univariate analysis with χ^2^ and Fisher’s exact tests was used to determine the association between seropositivity and age (years), sex, and ECG changes. A multivariate analysis was then performed to calculate the odd ratios between *T. cruzi* seropositivity and the risk factors.

**Results:**

The seropositive rate was 1.93% (52/2,688). In the ECG study, 22.85% (8/35) of the infected individuals exhibited ECG abnormalities. *Triatoma gerstaeckeri* was the only vector reported. The main risk factors were ceiling construction material (*P* ≤ 0.0024), domestic animals (*P* ≤ 0.0001), and living in rural municipalities (*P* ≤ 0.0025).

**Conclusions:**

These findings demonstrate a 10-fold higher prevalence of Chagas disease than previously reported (0.2%), which implies a serious public health threat in northeastern Mexico. The epidemiological profile established in this study differs from that found in the rest of Mexico, where human populations live in close proximity to domiciliary triatomines.

## Background

Chagas disease, or American trypanosomiasis, is a major disease and public health issue caused by the protozoan parasite *Trypanosoma cruzi*. This parasitic disease is widely distributed from South America northward to Texas in the United States [1]. An estimated 9–12 million people are currently infected, and approximately 60 million people are at risk of infection [2]. *T. cruzi* is transmitted by “kissing bugs” (Family: Reduviidae), insects with life-cycle stages that include domestic, peridomestic, and zoonotic cycles [3]. *T. cruzi* can also be transmitted by blood transfusion and congenital infection [4].

Mexico is a country with a wide variety of climates and significant biodiversity, where at least 30 species of triatomine bugs are recognized as vectors of Chagas disease [5]. In the rural areas of Nuevo Leon (northeastern Mexico), there have been few reports of the prevalence of wild reservoirs and vectors of *T. cruzi* since 1947 [6,7]. An important update was recently released [8] on the seroprevalence of *T. cruzi* in blood donors, highlighting the migration of seropositive individuals from endemic to nonendemic states as a main cause of subsequent *T. cruzi* infection. However, the current prevalence of *T. cruzi* infection in northeastern Mexico (especially in rural areas) has not been fully documented, and the few existing reports that address this issue in Nuevo Leon are limited. The National Seroepidemiological Survey (NSS), conducted by the office of the Secretaria de Salud, reported a seroprevalence of 0.2% in 1992 [9]. The aims of the present study were to (1) estimate the current seroprevalence rate of anti-*T. cruzi* antibodies, (2) analyze the risk factors associated with infection, (3) gather entomological data on the vectors of *T. cruzi*, and (4) collect electrocardiographic (ECG) data on infected individuals in 10 municipalities considered representative of the state of Nuevo Leon.

## Methods

### Study areas

**Figure 1:**
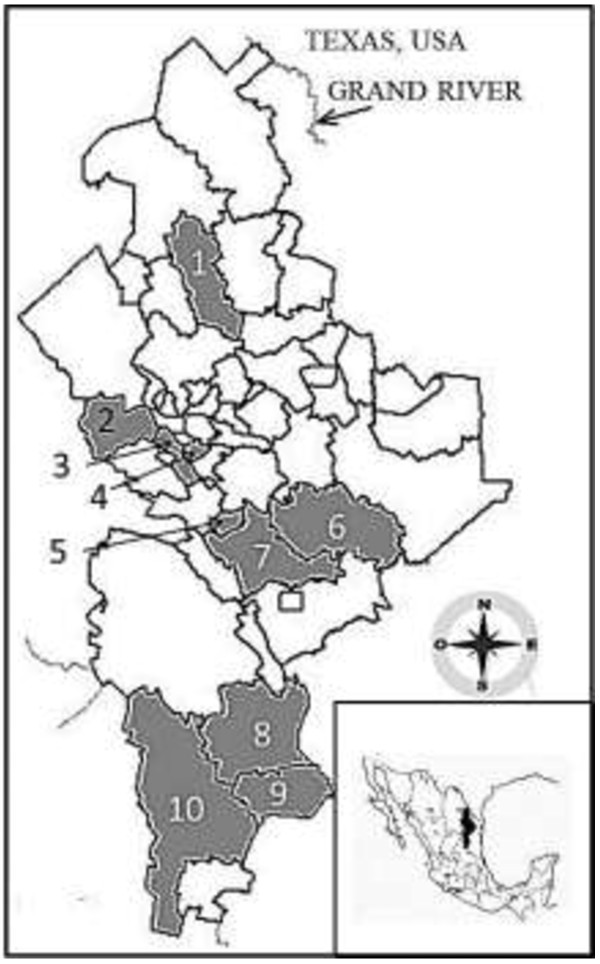
**Study site.** The smaller map (bottom right) identifies the state of Nuevo Leon situated in northeast Mexico; the Rio Grande is the border with the United States of America (Texas). The main map shows the locations of the municipalities studied (gray areas). 1: Sabinas Hidalgo; 2: Garcia; 3: Monterrey, 4: Guadalupe; 5: Allende; 6: General Teran; 7: Montemorelos; 8: Aramberri; 9: General Zaragoza; and 10: Doctor Arroyo.

Nuevo Leon, located in northeastern Mexico, shares its northern border with the state of Texas, USA (27°49‘N, 23°11’S and 98°26‘E, 101°14‘W). This study was conducted from April 2007 to September 2011 in 10 municipalities randomly selected from the 51 municipalities of the state of Nuevo Leon (including Monterrey, the capital; Figure 1). The altitude of the study area ranges from 90 to 3,710 m above sea level (MASL). The main economic activities in the urban zones (Guadalupe, Monterrey, and Garcia) are industry, commerce, and education. In the suburban regions (Sabinas Hidalgo, Allende, and Montemorelos), the main activities are citrus agriculture, juice industry, livestock, and chicken farming. In the rural localities (Arramberri, General Teran, General Zaragoza, and Doctor Arroyo), corn, watermelon, and sorghum agriculture predominates, although some residents also cultivate lechuguilla (*Agave lechuguilla*), which is used to make fiber for ropes and carpets [10].

### Study population

The sample size required was calculated based on an estimated prevalence of *T. cruzi* antibodies of 0.2%, reported by the NSS [9]. Nuevo Leon has a population of 4,653,458 inhabitants, and a sample group of 2,688 individuals was analyzed (Table 1), exceeding the calculated sample size required (384), with an absolute precision of 1.96, a confidence level of 95%, and *E* = 5%.

**Table 1:** Sample size by municipalities with results of entomological survey and seroprevalence by gender

**Municipality**	**Sample size**		**Recognition of vector**	**Seropositive subjects by ELISA and HAI**		
**Urban area**	**Houses**	**Subjects**	***N*/%**	**Male [positive/total (%)]**	**Female [positive/total (%)]**	**Total/%**
Garcia	21	50	2/4	0/18	0/32	0
Monterrey	154	556	9/1.6	3/244 (1.2)	5/312 (1.6)	8/1.4
Guadalupe	85	643	6/0.9	1/206 (0.4)	3/437 (0.6)	4/0.6
Subtotal	260	1249	17/1.36	4/468 (0.85)	8/781 (1.02)	12/1249 (0.96)
Suburban area						
Allende	75	380	9/2.3	4/177 (2.2)	6/203 (2.9)	10/2.6
Sabinas Hidalgo	20	100	2/2	1/49 (2)	1/51 (1.9)	2/2
Montemorelos	52	311	5/1.6	4/133 (3.0)	3/178 (1.6)	7/2.2
Subtotal	147	791	16/2.02	9/359(2.5)	10/432 (2.31)	19/2.4
Rural area						
Aramberri	56	143	4/2.79	1/61 (1.6)	0/82	1/0.6
General Teran	34	113	23/20.3	3/42 (7.1)	7/71 (9.8)	10/8.8
General Zaragoza	47	112	5/4.46	2/41 (4.8)	1/71 (1.4)	3/2.6
Doctor Arroyo	42	280	18/6.42	3/124 (2.4)	4/156 (2.5)	7/2.5
Subtotal	179	648	50/7.71	9/268 (3.35)	12/380 (3.15)	21/(3.2)
Total	586	2688	83/(3.0)	22/1095 (2.0)	30/1593 (1.88)	52/1.93

N: Number of inhabitants with knowledge of triatomines inside or outside the house. %: Prevalence.

### Epidemiological survey

The statistical sample was designed according to a stratified model. Systematic random-stratified household sampling was performed, based on the total number of inhabitants distributed in 51 municipalities of the state, and 10 localities were randomly selected. This model allows one to represent rural, suburban, and urban regions according to the socioeconomic and cultural information reported by the Instituto Nacional de Estadística y Geografía (INEGI) [10]. The urban population was selected to include houses with complete sanitary services (drinking water, sanitary drainage), commercial centers, hospitals, schools, and public transport. The suburban population included individuals living at the peripheries of cities. The rural population was characterized as living in localities with unpaved roads, without street lights or sanitary services, and with the presence of domestic and peridomestic animals (present in hen houses, pig pens, stables, etc.). Poor and well-built housing were equally represented in each region, but in rural and suburban areas, housing was constructed with adobe. A socioeconomic index was developed using household characteristics as the variables recorded: wall, roof, and floor materials, piped water, number of rooms. These were assigned numerical values of 0, 1, or 2, representing three economic strata. Between two and 10 housing blocks were selected for sampling from maps [10], according the density of each population. The selection procedure for the houses to be sampled involved the enumeration of each house in the block, and the selection of five houses on each side, with the previous consent of the householder.

Participation in the study was random and voluntary, with the sample size required obtained in each locality with no preference for sex, age, occupation, or residency. The epidemiological survey focused on determining the risk factors associated with the presence of triatomine bugs in the household and infection with *T. cruzi*. The questionnaire ascertained information on place of birth, age, sex, and years of residence in Nuevo Leon, because some individuals reported their original residence to have been in other municipalities or states, and that they had come to Nuevo Leon in search of employment or opportunities to travel to the United States. Another set of questions (independent variables) was designed according to the official national methodology for the surveillance, prevention, and control of vector-transmitted diseases [11] to evaluate the household characteristics, pet ownership (dogs and cats), and knowledge of triatomine bugs (representative mounted specimens from the locality were shown).

With the consent of the participants, blood samples were collected in Vacutainers by physicians (Daniel P. Molina Garza) and nurses. The sera were separated by centrifugation (1200 × *g* for 10 min), and the samples were stored at -20°C until analysis [8].

### Triatomine sampling

An entomological search for triatomine bugs was performed in the domestic and peridomestic environments for 30–60 min, with the involvement of members of the community. Inside the houses, the wall cracks, roofs, spaces beneath beds, piles of wood, and areas around the tables and chairs in the kitchens were searched. Outside the houses, piles of wood or stone, wooden fences, and wall slits [11] were also searched. In the evening, a light trap device was used outside the houses. Triatomines were identified according to the criteria of Lent and Wygodzinsky [12].

### Enzyme-linked immunosorbent assay (ELISA)

An ELISA (Chagatest ELISA recombinant v. 3.0, Wiener Lab Group, Rosario, Argentina) was performed, following the manufacturer’s protocols. The absorbance of the samples was measured spectrophotometrically at 450/620 nm (Multiscan MS, Thermo Labsystems, Waltham, MA). Each test was performed in duplicate. Positive sera from chronic chagasic patients from Brazil and Mexico, provided by the Instituto Nacional de Cardiología Ignacio Chavez, D.F., Mexico, and negative sera from healthy individuals were used as the controls for each test. The cut-off (CO) value was calculated according to the manufacturer’s instructions using the equation CO = NC + 0.3 OD, where NC = the average absorbance of the negative controls and OD = optical density.

### Indirect hemagglutination analysis (IHA)

IHA (Serodia-Chagas, Fujirebio Inc., Tokyo, Japan) was performed according to the manufacturer’s instructions. Reactive samples at a dilution of ≥ 1:32 were considered positive. All samples were analyzed in duplicate, including the positive and negative control sera.

### ECG study

An ECG case–control study was performed with a 12-derivation electrocardiograph (Bionet, Cardiocare 2000, Korea) at a paper speed of 10 mm/s. Volunteers with a positive serology for *T. cruzi* were matched by sex and age to seronegative residents of the same locality. The results were analyzed and interpreted by blinded evaluators, and the ECG results (normal or abnormal) were classified according to the deductive method of ECG interpretation and criteria by our medical personnel [1,13].

### Data management analysis

**Figure 2:**
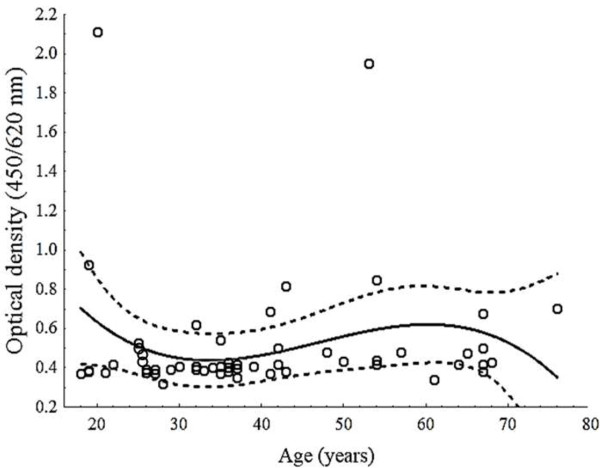
**Distribution of *Trypanosoma cruzi *infection in Nuevo Leon, Mexico, by ELISA according to age (circles).** Results of IHA were ≥ 1:32. Optical density results are shown as means ± 95% confidence intervals of a second-order polynomial regression, as indicated by the regression line and its 95% confidence area (dotted line).

Seropositive samples (ELISA and HAI) were recorded and plotted according to age by second-order polynomial regression with their 95% confidence intervals (CI; Figure 2). A univariate analysis with χ^2^ and Fisher’s exact tests was used to determine the association between seropositivity and age (years), sex, and ECG changes, with their 95% CIs (*P* ≤ 0.05). Multivariate logistic regression was then used to calculate the odds ratios (ORs) between *T. cruzi* seropositivity and the risk factors using the SPSS software, version 17 (Chicago, IL) and GraphPad Prism version 6.01. (La Joya, CA). Only variables significantly associated in the univariate regression were included.

### Ethical considerations

The study protocol was reviewed and approved by the Ethics Committee of the Universidad Autónoma de Nuevo León. According to the “Reglamento de la Ley General de Salud en Materia de Investigación”, forearm venipuncture was considered a minimal-risk procedure, requiring only verbal consent when unschooled participants are invited to participate in a clinical or epidemiological study.

## Results

A total of 2,688 serum samples from 1,095 men and 1,593 women from 586 dwellings located in rural, suburban, and urban localities in 10 municipalities of Nuevo Leon were analyzed (Table 1). Except for four participants aged 18–19 years (Figure 2), we were unable to enroll young participants. Of the samples analyzed, 52 (1.93%) were positive for *T. cruzi* antibodies on ELISA and IHA serological tests (Tables 1 and 2, respectively). Antibodies against *T. cruzi* were identified in nine municipalities. The prevalence rates differed among the urban (0.96%, 12/1249), suburban (2.4%, 19/791), and rural areas (3.2%, 21/648).

**Table 2:** Age and gender distribution of seropositive subjects by age groups

**Age (years)**	**Male [positive/total (%)]**	**Female [positive/total (%)]**	**Total [positive/total (%)]**
17-27	7/452 (1.54)	10/585 (1.7)	17/1037 (1.63)
28-36	4/326 (1.22)	7/365 (1.91)	11/691 (1.59)
37-53	6/137 (4.37)	8/293 (2.73)	14/430 (3.25)
54-79	5/180 (2.77)	5/350 (1.42)	10/530 (1.88)
Total	22/1095 (2.0)	30/1593 (1.88)	52/2688 (1.93)

Seroprevalence were statistically significant between age and gender groups (Chi-square = 44.36, *P* ≤ 0.0001).

### Epidemiological survey

The seropositive rates were not significantly dependent on sex or age (χ^2^ = 3.28, *P* = 0.351; and χ^2^ = 5.430, *P* = 0.349, respectively), although men had a slightly higher infection rate (2%) than women (1.88%). The epidemiological survey showed that 83 participants (3.0%) were able to identify triatomine bugs, and most of these individuals (50, 60.24%) lived in rural zones. Although the participants from General Teran were better able to identify triatomines inside or around their dwellings, seropositivity was higher in this municipality (10/113, 8.8%; Table 1). Populations from urban areas were unfamiliar with the vector, and few were able to identify triatomines as hematophagous insects (Table 1). Lower antibody titers were observed in women from urban areas than in women from rural areas (data not shown). The infection rate in women of child-bearing age (17–45 years old) was 40.38%, representing 21 of the 52 seropositive individuals (data not shown) and 1.31% of the female population sampled (21/1593, 95% CI = 0.88–2.44).

Only two adult triatomine bugs (*Triatoma gerstaeckeri*), both negative for *T. cruzi*, were collected in the urban municipality of Guadalupe. Because nine of the municipalities were negative for the presence of the vector, the entomological index was not calculated.

We found that *T. cruzi* infection was strongly associated with ECG abnormalities (χ^2^ = 9.243, *P* = 0.002, Table 3). ECG results were obtained from 35 of the 52 seropositive participants; 27 of the ECG scans were normal and eight were abnormal (8/35, 22.85%). Most of the abnormal ECG data showed a high prevalence of right bundle branch hemiblock (RBBHB; 5/8, 62.5%), whereas only two seronegative controls had this abnormality (2/9; 22.2%; Table 3). Seventeen participants could not be located; surveys showed that they had returned to their work as immigrants in the United States or other urban areas in the state. A statistical analysis showed a significant correlation between abnormal ECG findings and age *(*χ^2^ = 27.93, *P* = 0.0001), regardless of whether the participants who were lost to follow-up were included (χ^2^ = 20.75, *P* = 0.00001). Abnormal ECG findings correlated significantly with female sex (χ^2^ = 17.500, *P* = 0.01), and patients with abnormal ECG readings represented 62.5% of cases (5/8), as shown in Table 3.

**Table 3:** Electrocardiogram (ECG) findings in seropositive and seronegative volunteers

	**Abnormal ECG**	
	** Seropositive**	** Seronegative**
Community	N°/ [Total of seropositive (%)]	N°/ [Total of seronegative (%)]
	Sex/Age‡/ECG alteration	Sex/Age‡/ECG alteration
Monterrey	1/8 (12.5)	3/8 (37.5)
	F/54/RBBHB	F/55/AF, M/57/ST, F/56/ST
Allende	1/10 (10)	2/10 (20)
	M/57/RBBHB	M/58/AF, M/59/RBBHB
General Teran	4/10 (40)	3/10 (30)
	M/53/RBBHB M/67/RBBHB,	M/56/LBBHB, M/65/RBBHB
	F/65/LBBHB, F/68/RBBB	F/65/ST, F/67/ST
Doctor Arroyo	2/7 (28.5)	1/7 (14.2)
	F/54/LBBB, F/61/RBBHB	F/55/AF
Total^†^	8/35 (22.8)	9/35 (25.7)

^†^*χ*^*2*^ comparing ECG of seropositive and seronegative groups (*P* = 0.002) , and ‡ age (*P* = 0.0001) were significant. AF = Atrial fibrillation; LBBB = Left bundle branch block; LBBHB = Left bundle branch hemiblock; RBBHB = Right bundle branch hemiblock; RBBB = Right bundle branch block; ST = Sinus tachycardia.

The frequency of each variable evaluated as a possible risk factor and the corresponding ORs for the associations between the risk factors and seropositivity are shown in Table 4. Of the 52 seropositive individuals, 40 had pets or were involved in animal breeding (horses, pigs, sheep, or poultry), and a positive association was observed between infection and the presence of animals (OR = 6.73, 95% CI = 3.15–4.39, *P* = 0.0001) or certain ceiling construction materials (palm leaves and curved lamina tile: OR = 2.52, 95% CI = 1.41–4.51, *P* ≤ 0.0024; Table 4). Overall, 21 seropositive individuals (40.38%) were detected in rural areas, and the association with animals was significantly higher (OR = 3.09, 95% CI = 1.48–6.47, *P* ≤ 0.0025) than in urban or suburban areas (OR = 2.30, 95% CI = 1.08–4.85, *P* = 0.036), where 19 and 12 seropositive subjects were detected, respectively.

**Table 4:** **R** **isk factors analyzed with seropositive inhabitants and household characteristics**

**Variable (household characteristics)**	**N° houses**	**Seropositive subjects**	**Frequency (%)**	**OR**	**95% CI**	***P *** **value**
Birthplace						
Nuevo Leon vs. other	459/127	43/9	9.36/7.08	0.75	0.35-1.59	0.5961*
Flooring construction material						
Concrete vs. dirt	584/2	51/1	8.73/50	5.72	0.51-64.26	0.2255*
Walls construction material						
Concrete vs. adobe brick	462/124	37/15	8/12.09	1.51	0.80-2.84	0.2194*
Ceiling construction material Concrete vs. palm leaves and curve lamina tile	446/140	29/23	6.50/16.42	2.52	1.41-4.51	0.0024^‡^
Domestic pets and livestock						
Presence vs. absence	561/25	40/12	7.13/48	6.73	3.15-4.39	≤ 0.0001^†^
Variable (Residential place of seropositive individuals)						
Urban vs suburban	260/179	12/19	4.61/10.61	2.30	1.08-4.85	0.036^‡^
Urban vs. rural	260/147	12/21	4.61/14.28	3.09	1.48-6.47	0.0025^‡^

*Not significant. ^‡^Significant. ^†^Highly significant.

## Discussion

The participants in this study were from a non-*T. cruzi*-endemic state (Nuevo Leon) in northeastern Mexico, for which little information on Chagas disease is available. Previously, we reported the prevalence of seropositive blood donors (2.8%) in northeastern Mexico [8] and stressed the need to update the existing knowledge about Chagas disease in an unrestricted population from this region. In this study, we have not only updated the prevalence of anti-*T. cruzi* antibodies in the inhabitants of urban, suburban, and rural municipalities in this region, but have also identified the epidemiological features associated with positive serology (including ECG abnormalities) and have shown a significant correlation between ECG abnormalities and age. Our results demonstrate that the seroprevalence of *T. cruzi* infection (1.93%) in Nuevo Leon is higher than that reported in previous studies of the people from this state (0.2%), conducted between 1987 and 1989 by the NSS [9]. In general, our results show that there has been an increase of around 10-fold in the prevalence of Chagas disease in Nuevo Leon. Recent reports have shown similar results, with higher seroprevalence rates than previously reported [9] for states in central (Morelos, Puebla, Hidalgo, Veracruz, San Luis Potosi, and Colima) [14-19] and southern Mexico (Chiapas and Yucatan) [20-22]. Therefore, it appears that the prevalence of *T. cruzi* is increasing throughout the state of Nuevo Leon [8,9].

Women of child-bearing age had a seroprevalence rate of 1.31% (data not shown), which suggests that congenital transmission may contribute to the elevated infection rate in children. Therefore, future research should focus on the investigation of congenital transmission. The INEGI recorded a population of 1,041,600 women of reproductive age and 93,902 births in Nuevo Leon in 2010 [10]. Extrapolating from the highest national seroprevalence reported for *T. cruzi*[4,5], we calculated that up to 61,454 women of child-bearing age may be infected and 5,540 infants congenitally infected in Nuevo Leon. This situation requires the urgent establishment of an active surveillance program to determine the major mechanisms of transmission and the incidence of infections acquired through blood transfusion. Programs to screen for congenital Chagas disease transmission are also needed.

Our findings reveal several important features of the epidemiology of *T. cruzi* infection in Nuevo Leon. We found that seropositivity did not increase significantly with age. However, 28 seropositive participants (53.84%) were younger than 37 years (Figure 2, Table 2), a rate lower than that previously reported by other studies [9,16 ]. In this study, it was not possible to analyze sera from anyone under the age of 17 years, although two participants from General Teran who were 18 and 19 years of age showed higher antibody titers than the older participants (Figure 2), suggesting that further studies are necessary to determine whether active vector transmission exists. In comparison, in Veracruz [17] and San Luis Potosi [18], two neighboring states located southeast and south of Nuevo Leon, respectively, the *T. cruzi* infection rates among children 5–14 and 10 years old, respectively, were recently been reported. In this study, we found that living in a rural area, specific ceiling construction materials, and domestic pets and livestock are the main risk factors associated with the seroprevalence rate. Our results confirm previous reports identifying these risk factors [2,23,24].

Ecological features also appeared to be significantly associated with the seroprevalence of *T. cruzi*[21], and a higher seroprevalence was observed in municipalities located at 310–460 MASL [5] (General Teran and Allende). The behavior of *T. gerstaeckeri*, a predominantly peridomiciliary and wild vector, also influenced the infection rates [5,7,12,25]. This is similar to the profile reported in the state of Texas, USA, where up to 267,000 endemic (locally acquired) cases of Chagas disease were predominantly transmitted by the *T. gerstaeckeri* vector since 1995 [26,27]. Thus, the epidemiological profile found in this study differs from that of the rest of Mexico and Latin America, where human populations live in close proximity to domiciliary triatomines (*T. barberi*, *T. dimidiata*, *T. mexicana*, *T. phyllosoma*, and *T. longipennis*) [14,16-21,28,29].

We have demonstrated an association between seropositivity for *T. cruzi* infection and abnormal ECG findings, and similar associations have been observed in other states of Mexico [14,16]. Overall, 27 seropositive individuals showed normal ECG data, whereas eight had ECG abnormalities, particularly among individuals aged 54–68 years old. Five of the abnormal ECGs showed signs of RBBHB, the most characteristic finding of seropositive individuals with typical chagasic cardiomyopathies in Mexico and South America [1,20]. Our results indicate that both *T. cruzi* infection and Chagas disease are present in Nuevo Leon. It is probable that many cases of Chagas disease remain unrecognized. It is less likely that circulating strains of *T. cruzi* in some regions of Mexico are less virulent [14,15,30] (i.e., mainly silent) than those circulating in other parts of Latin America. In Nuevo Leon, Chagas disease remains poorly recognized, and most physicians consider it to be an “exotic” disease, exclusive to South America [9,31].

This study had some limitations. First, the prevalence bias could have been underestimated because the sample size was calculated for a seroprevalence rate of 0.2%, reported by the NSS [9] from an IHA/IFI test at titers of 1:32. However, no sensibility data were reported. The sample used by the NSS included 3,747 inhabitants, but the origins of the samples (rural, suburban, and urban counties) were not reported, which might have caused us to underestimate the prevalence in the present study. Second, sample size is one of the major limitations in most epidemiological studies. The statistical power of a study to assess epidemiological associations depends on the number of participants, in this case 2,688 individuals. However, it was not possible to obtain uniformity in the subsample sizes according to age, consistent with our study design, and a major defect of the study was our failure to sample individuals < 17 years old, because the samples were obtained with the voluntary participation of the population, and if children are included, the consent of their parents is legally required. Another limitation was the high mobility of the immigrant population, from which 17 seropositive participants were lost to follow-up and consequently to ECG analysis, reducing the effective number of participants. Finally, the participation of the community members in identifying triatomines was low (3%); only 83 individuals correctly identified the specimens of *Triatoma* adults shown to them, although the major difficulty was in recognizing the nymphs (often confused with phytophagous insects). In Doctor Arroyo, triatomines were recognized only by older individuals, which was also a limitation of the epidemiological survey.

## Conclusions

In conclusion, this study has established the seroprevalence of specific anti*-T. cruzi* antibodies (1.93%; 52/2,688) and demonstrated an association between seropositivity and ECG abnormalities (RBBHB) in healthy volunteers living in Nuevo Leon. We also identified specific ceiling construction materials, domestic animals, and living in rural municipalities as important risk factors for *T. cruzi* infection. These findings have important implications in northeastern Mexico, identifying *T. cruzi*-transmitted Chagas disease as a serious public health threat.

## Abbreviations

ELISA: Enzyme-linked immunosorbent assay; IHA: Indirect hemagglutination analysis; ECG: Electrocardiographic; OR: Odd ratios; CI: Confidence interval.

## Competing interests

The authors declare that they have no competing interests.

## Authors’ contributions

ZJMG conceived the study, performed the serological analyses, analyzed the data, and wrote the manuscript. JLRE wrote the protocol and reviewed the analyses. RMH performed the statistical analyses and proofread the manuscript. DPMG, gathered the samples and performed the ECG study. RGF developed the proposal, participated in the study design, and edited the article. LGS conceived the study, participated in sample collection, participated in the coordination and management of the study, collected and analyzed the data, and edited the article. All authors read and approved the final manuscript.

## Pre-publication history

The pre-publication history for this paper can be accessed here:

http://www.biomedcentral.com/1471-2334/14/117/prepub
